# CHOP versus GEM-P in previously untreated patients with peripheral T-cell lymphoma (CHEMO-T): a phase 2, multicentre, randomised, open-label trial

**DOI:** 10.1016/S2352-3026(18)30039-5

**Published:** 2018-04-24

**Authors:** Mary Gleeson, Clare Peckitt, Ye Mong To, Laurice Edwards, Jacqueline Oates, Andrew Wotherspoon, Ayoma D Attygalle, Imene Zerizer, Bhupinder Sharma, Sue Chua, Ruwaida Begum, Ian Chau, Peter Johnson, Kirit M Ardeshna, Eliza A Hawkes, Marian P Macheta, Graham P Collins, John Radford, Adam Forbes, Alistair Hart, Silvia Montoto, Pamela McKay, Kim Benstead, Nicholas Morley, Nagesh Kalakonda, Yasmin Hasan, Deborah Turner, David Cunningham

**Affiliations:** a The Royal Marsden NHS Foundation Trust, London and Surrey, UK; b Cancer Research UK Centre, University of Southampton, Southampton, UK; c University College Hospital London, London, UK; d Olivia Newton John Cancer Research Institute, Austin Health, Melbourne, VA, Australia; e Eastern Health, Melbourne, VA, Australia; f Blackpool Teaching Hospitals, Blackpool, UK; g Oxford Cancer and Haematology Centre, Churchill Hospital, Oxford, UK; h University of Manchester and the Christie NHS Foundation Trust, Manchester Academic Health Science Centre, Manchester, UK; i Royal Cornwall Hospital, Truro, UK; j New Victoria Hospital, Glasgow, UK; k St Bartholomew's Hospital, London, UK; l Beatson West of Scotland Cancer Centre, Glasgow, UK; m Gloucestershire Hospitals NHS Foundation Trust, Gloucestershire, UK; n Sheffield Teaching Hospitals NHS Foundation Trust, Sheffield, UK; o University of Liverpool, Liverpool, UK; p Sandwell and West Birmingham Hospitals NHS Trust, Birmingham, UK; q Torbay and South Devon NHS Trust, Torquay, UK

## Abstract

**Background:**

Outcomes with CHOP (cyclophosphamide, doxorubicin, vincristine, and prednisolone) or CHOP-like chemotherapy in peripheral T-cell lymphoma are poor. We investigated whether the regimen of gemcitabine, cisplatin, and methylprednisolone (GEM-P) was superior to CHOP as front-line therapy in previously untreated patients.

**Methods:**

We did a phase 2, parallel-group, multicentre, open-label randomised trial in 47 hospitals: 46 in the UK and one in Australia. Participants were patients aged 18 years and older with bulky (tumour mass diameter >10 cm) stage I to stage IV disease (WHO performance status 0–3), previously untreated peripheral T-cell lymphoma not otherwise specified, angioimmunoblastic T-cell lymphoma, anaplastic lymphoma kinase-negative anaplastic large cell lymphoma, enteropathy-associated T-cell lymphoma, or hepatosplenic γδ T-cell lymphoma. We randomly assigned patients (1:1) stratified by subtype of peripheral T-cell lymphoma and international prognostic index to either CHOP (intravenous cyclophosphamide 750 mg/m^2^, doxorubicin 50 mg/m^2^, and vincristine 1·4 mg/m^2^ [maximum 2 mg] on day 1, and oral prednisolone 100 mg on days 1–5) every 21 days for six cycles; or GEM-P (intravenous gemcitabine 1000 mg/m^2^ on days 1, 8, and 15, cisplatin 100 mg/m^2^ on day 15, and oral or intravenous methylprednisolone 1000 mg on days 1–5) every 28 days for four cycles. The primary endpoint was the proportion of patients with a CT-based complete response or unconfirmed complete response on completion of study chemotherapy, to detect a 20% superiority of GEM-P compared with CHOP, assessed in all patients who received at least one cycle of treatment and had an end-of-treatment CT scan or reported clinical progression as the reason for stopping trial treatment. Safety was assessed in all patients who received at least one dose of study medication. This trial is registered with ClinicalTrials.gov (NCT01719835) and the European Clinical Trials Database (EudraCT 2011-004146-18).

**Findings:**

Between June 18, 2012, and Nov 16, 2016, we randomly assigned 87 patients to treatment, 43 to CHOP and 44 to GEM-P. A planned unmasked review of efficacy data by the independent data monitoring committee in November, 2016, showed that the number of patients with a confirmed or unconfirmed complete response with GEM-P was non-significantly inferior compared with CHOP and the trial was closed early. At a median follow-up of 27·4 months (IQR 16·6–38·4), 23 patients (62%) of 37 assessable patients assigned to CHOP had achieved a complete response or unconfirmed complete response compared with 17 (46%) of 37 assigned to GEM-P (odds ratio 0·52, 95% CI 0·21–1·31; p=0·164). The most common adverse events of grade 3 or worse in both groups were neutropenia (17 [40%] with CHOP and nine [20%] with GEM-P), thrombocytopenia (4 [10%] with CHOP and 13 [30%] with GEM-P, and febrile neutropenia (12 [29%] with CHOP and 3 [7%] with GEM-P). Two patients (5%) died during the study, both in the GEM-P group, from lung infections.

**Interpretation:**

The number of patients with a complete response or unconfirmed complete response did not differ between the groups, indicating that GEM-P was not superior for this outcome. CHOP should therefore remain the reference regimen for previously untreated peripheral T-cell lymphoma.

**Funding:**

Bloodwise and the UK National Institute of Health Research.

## Introduction

Peripheral T-cell lymphoma is a rare and heterogeneous subgroup of non-Hodgkin lymphomas, and comprises approximately 10% of all non-Hodgkin lymphomas in Europe and America.^1^ Randomised prospective trials in patients with peripheral T-cell lymphoma are scarce and hence there is no consensus on the optimal chemotherapy for previously untreated patients, although combination chemotherapy with cyclophosphamide, doxorubicin, vincristine, and prednisolone (CHOP) is widely used, with consolidative autologous stem-cell transplantation considered in eligible patients. However, for most patients, outcomes with CHOP are poor, with only 33% to 43% achieving a complete response^2^, ^3^, ^4^ and 38·5% achieving 5-year overall survival;^5^ therefore, a superior upfront regimen for peripheral T-cell lymphoma is urgently required. Previous evidence suggested that the addition of etoposide to CHOP (CHOEP) might improve event-free survival for younger patients (aged ≤60 years) without increased lactate dehydrogenase,^6^ but no benefit for overall survival was apparent and this approach is not widely applicable.

**Research in context**

**Evidence before this study**

CHOP combination chemotherapy (cyclophosphamide, doxorubicin, vincristine, and prednisolone) is widely used for treatment of peripheral T-cell lymphoma; however, outcomes with CHOP are poor for most patients. We investigated GEM-P chemotherapy (gemcitabine, methylprednisolone, and cisplatin) compared with CHOP in previously untreated patients with peripheral T-cell lymphoma. We searched PubMed on Dec 4, 2017, for English-language articles published from January, 1998, to October, 2017, and searched abstracts from the American Society of Hematology and American Society of Clinical Oncology published between 2015–17 with the search terms “T-cell lymphoma”, “chemotherapy”, and “gemcitabine”, excluding studies in which patients with non-nodal peripheral T-cell lymphoma forms were assessed exclusively. We identified 30 reports showing activity of gemcitabine in peripheral T-cell lymphoma; three with gemcitabine as monotherapy and nine with a combination of gemcitabine with platinum and steroids predominantly in pretreated populations, including three retrospective reports on GEM-P specifically in peripheral T-cell lymphoma. Different combinations of gemcitabine with other novel drugs, with or without the addition of platinum, in the treatment of peripheral T-cell lymphoma were also reported in the scientific literature, including one randomised trial that assessed the combination of gemcitabine, cisplatin, prednisolone and thalidomide versus CHOP in treatment-naive patients. However, there were no reported randomised studies on the combination of gemcitabine, platinum, and steroids alone versus CHOP in the front-line setting.

**Added value of this study**

Our phase 2 trial is one of the few randomised studies in previously untreated patients with peripheral T-cell lymphoma and is an important addition to the evidence-base. The findings confirm the poor outcomes for peripheral T-cell lymphoma within a prospective trial cohort and indicate that CHOP should remain the reference regimen at present. Our trial was the first prospective study to assess an ^18^F-FDG-PET-CT response in patients with peripheral T-cell lymphoma as part of a pre-planned substudy, and the data suggest that ^18^F-FDG-PET-CT might be a more sensitive tool than contrast-enhanced CT for determining response in peripheral T-cell lymphoma, as it might better distinguish between a residual fibrotic mass present after chemotherapy versus viable tumour. Furthermore, obtaining a complete response by ^18^F-FDG-PET-CT was independently prognostic for superior progression-free survival in multivariable analysis, whereas complete response by CT was not. Additionally, we reported the incidence and pattern of CNS relapse in peripheral T-cell lymphoma from a prospective trial. Determination of the subtype of peripheral T-cell lymphoma in our study was revised for around a fifth of the patients, highlighting the diagnostic challenges in peripheral T-cell lymphoma. Despite GEM-P showing non-significant inferiority for the endpoint of CT-based confirmed and unconfirmed complete response compared with CHOP, there were no differences in either progression-free survival or overall survival between the groups; future study design in this indication should be powered for primary endpoints of survival rather than complete response, which might not be an accurate surrogate endpoint (particularly when assessed by contrast-enhanced CT) in peripheral T-cell lymphoma.

**Implications of all the available evidence**

Taken together, current evidence supports the use of gemcitabine as an effective therapy in the management of peripheral T-cell lymphoma. However, our randomised phase 2 study did not suggest superiority of GEM-P over CHOP in the front-line setting for previously untreated patients and therefore, CHOP should remain the reference regimen in this indication for previously untreated patients. Although GEM-P has shown efficacy in patients with peripheral T-cell lymphoma, at present it should be reserved for patients with relapsed or refractory disease. A superior upfront induction regimen is urgently required for patients with treatment-naive peripheral T-cell lymphoma, and future incorporation of novel drugs could enhance the efficacy of front-line therapy in this indication. Trials are ongoing (ClinicalTrials.gov NCT01777152, NCT01796002, and NCT02561273) to address this research question.

The nucleoside analogue gemcitabine is not effluxed by the multidrug resistance gene-1–P glycoprotein (MDR-1–Pgp), which is overexpressed in some peripheral T-cell lymphomas^7^, ^8^ on the tumour cells, residual lymphocytes, or in the endothelium^8^ and has shown activity both as monotherapy^9^, ^10^, ^11^ and in combination with platinum and steroids^12^, ^13^, ^14^, ^15^, ^16^, ^17^, ^18^, ^19^, ^20^ in patients with relapsed or refractory peripheral T-cell lymphoma. The regimen of intravenous gemcitabine 1000 mg/m^2^ on days 1, 8, and 15 of a cycle, intravenous cisplatin 100 mg/m^2^ on day 15, and oral or intravenous methylprednisolone 1000 mg on days 1–5 (GEM-P) administered every 28 days is associated with 69% to 100% of pretreated patients with peripheral T-cell lymphoma achieving an objective response, and 19% to 50% achieving a complete response;^14^, ^15^, ^16^ a median progression-free survival of 12 months has been reported in the largest retrospective series.^16^

Given the poor outcomes associated with CHOP as front-line therapy in peripheral T-cell lymphoma and the promising data in relapsing and refractory peripheral T-cell lymphoma with GEM-P, the UK National Cancer Research Institute (NCRI) Lymphoma Clinical Study Group started the CHEMO-T trial in 2012 to investigate the potential superiority of GEM-P compared with CHOP in previously untreated patients with peripheral T-cell lymphoma.

## Methods

### Study design and participants

We did a phase 2, parallel-group, multicentre, open-label randomised trial at 47 hospitals: 46 in the UK and one in Australia (appendix pp 1–2).

Eligible participants were patients aged 18 years and older with previously untreated histologically confirmed peripheral T-cell lymphoma of the WHO 2008 subtypes:^21^ peripheral T-cell lymphoma not otherwise specified, angioimmunoblastic T-cell lymphoma, anaplastic lymphoma kinase (ALK) negative anaplastic large cell T-cell lymphoma, enteropathy-associated T-cell lymphoma, and hepatosplenic γδ T-cell lymphoma.

For participation in the study, patients were required to have bulky stage I (tumour mass diameter >10 cm) to stage IV disease; a WHO performance status of 0–3 (patients with a performance status of 3 were only eligible if this was deemed to be related to a lymphoma); adequate cardiac, renal, hepatic, and bone marrow function (absolute neutrophil count ≥1·0 × 10^9^/L, white blood cell count ≥3·0 × 10^9^/L, platelet count ≥100·0 × 10^9^/L, and haemoglobin concentration ≥9·0 g/dL unless related to disease). Patients with CNS or leptomeningeal involvement, positive serology for HIV-1, active hepatitis B or C, or a history of malignancy within the preceding 5 years (with the exception of curatively treated skin cancers or carcinoma in situ of the cervix) and poorly controlled diabetes or other comorbidities which would preclude the safe delivery of treatment within the trial, were ineligible, as were patients without at least one site of measurable disease at baseline (measurable in two perpendicular dimensions and ≥1 cm on the longest diameter on contrast-enhanced CT scan, except for patients with enteropathy-associated T-cell lymphoma following complete surgical resection). Corticosteroids, not exceeding a maximum of prednisolone 100 mg/day (or equivalent corticosteroid dose) for 7 days, for symptoms related to lymphoma before starting study treatment were permitted, but preferably not instituted before an ^18^F-fluoro-deoxyglucose (FDG) PET-CT scan at baseline. If corticosteroids were instituted before the baseline scan, they were required to be discontinued 24 h beforehand and patients were required to have a serum glucose concentration of 8·0 mmol/L or lower immediately before the scan.

The protocol was approved by the UK Medicines and Healthcare products Regulatory Agency and London Riverside South West Research Ethics Committee. Ethics approval in Australia was from The Eastern Health Human Research Ethics Committee. Patients provided written informed consent.

### Randomisation

We assigned patients (1:1) to either CHOP or GEM-P. Randomisation was done centrally by the clinical trials unit at the Institute for Cancer Research (ICR) independently of the trial team and investigators using a minimisation procedure from the beginning of the trial and first patient, without a burn-in period. The stratification variables of locally-determined histological subtype and International Prognostic Index risk group (low 0–1 *vs* intermediate 2–3 *vs* high 4–5) were used for computer-based minimisation. We factored in a probability component (ie, 80% chance of an incoming patient being allocated to an unbalanced group). Given the differences in the administration schedules between the chemotherapy regimens under assessment, it was not possible to mask patients to the treatment they were receiving.

### Procedures

Patients in the CHOP group received cyclophosphamide 750 mg/m^2^, doxorubicin 50 mg/m^2^, and vincristine 1·4 mg/m^2^ (up to a maximum of 2 mg) intravenously on day 1, and oral prednisolone 100 mg on days 1–5 every 21 days, for six cycles. Patients in the GEM-P group received intravenous gemcitabine 1000 mg/m^2^ on days 1, 8, and 15, intravenous cisplatin 100 mg/m^2^ on day 15, and oral or intravenous methylprednisolone 1000 mg on days 1–5 every 28 days, for four cycles. The rationale for administering four cycles of GEM-P (rather than six cycles) was that because GEM-P is a platinum-based regimen used mainly for patients with relapsed or refractory disease, we anticipated that most patients would have achieved a maximum response with this type of regimen by the fourth cycle.

For both treatment groups, dose modifications were required for treatment-related toxicities in accordance with the study protocol. Dose banding as per local guidelines was permitted. Administration of supportive medication, including antiemetic therapy, prophylaxis for tumour lysis, granulocyte-colony stimulating factor (G-CSF), prophylaxis for *Pneumocystis jirovecii* pneumonia, fluid and electrolyte administration relating to cisplatin, and administration of prophylaxis for CNS relapse, was given in accordance with local practice.

Criteria for withdrawing a patient from study treatment were interruption of study treatment for more than 3 weeks for reasons of toxicity (or for >4 weeks for reasons other than toxicity), disease progression, withdrawal of consent, unacceptable treatment-related toxicity, pregnancy, patient non-compliance, or other events precluding further administration of study drugs as judged by the study chief investigator (DC).

On completion of study treatment, patients with a complete response or unconfirmed complete response could proceed to high-dose chemotherapy and autologous stem-cell transplantation at the local investigator's discretion. Consolidation radiotherapy to sites of initial bulk or residual disease was also permitted at the end of treatment at the investigator's discretion.

Patients were assessed at baseline, at each attendance for treatment, 30 days after completion of treatment, and subsequently every 3 months for 1 year, then every 6 months until year 2, and every year thereafter for a maximum follow-up of 5 years. Laboratory monitoring with a full blood-cell count (haemoglobin, white cells, neutrophils, and platelets) and biochemistry (sodium, potassium, urea, creatinine, calcium, magnesium, phosphate, lactate dehydrogenase, total bilirubin, liver transaminases, alkaline phosphatase, serum albumin, and blood glucose concentrations) were done at baseline, on each day of treatment, and at 30 days after completion of study treatment. At baseline all patients had a contrast enhanced CT scan of the neck, thorax, abdomen and pelvis and an ^18^F-FDG-PET-CT scan done within 28 days of randomisation, and a bone marrow biopsy was required within 6 weeks of randomisation. CT scans were also required during treatment (after cycles 2 and 4 for patients on CHOP and after cycles 1 and 3 for patients on GEM-P). Both a contrast enhanced CT scan and an ^18^F-FDG-PET-CT scan were done at the end of treatment. Any clinical suspicion of relapse or progression was confirmed radiologically or on bone marrow aspirate or trephine if disease was marrow-based only.

All patients enrolled were required to submit their diagnostic tissue for central histopathology review within 28 days of randomisation; CT and ^18^F-FDG-PET-CT responses were also centrally assessed. Site accreditation, data collation, and quality control for ^18^F-FDG-PET-CT imaging was done by the UK PET Core Lab (London, UK).

### Outcomes

The primary endpoint of the trial was the proportion of patients who achieved an investigator-assessed, CT-based complete response or unconfirmed complete response on completion of study chemotherapy, according to the International Workshop Standardized Response Criteria for non-Hodgkin lymphoma.^22^

The primary endpoint was locally assessed and analysed as randomised in the assessable population, defined as all eligible patients who received at least one cycle of treatment and had an end of treatment scan or reported clinical progression as a reason for stopping trial treatment. A patient with no CT imaging but a report of clinical progression as reason for stopping trial treatment was categorised as having progressed.

A preplanned primary endpoint sensitivity analysis was done in the intention-to-treat (ITT) population including all eligible patients who received at least one cycle of treatment; patients with no end-of-treatment-CT assessment were counted as non-responders. A second preplanned primary endpoint sensitivity analysis was done as treated in the per-protocol population defined as all patients who received the planned number of cycles and were either assessable by CT or had reported clinical progression at the end of treatment.

The secondary endpoints of the trial were overall survival, progression-free survival, partial response, stable disease, progressive disease, and toxicity, and the proportion of patients with metabolic complete response determined by ^18^F-FDG-PET-CT, according to the Revised Response Criteria for Malignant Lymphoma.^23^ A secondary endpoint assessing ^18^F-FDG-PET-CT response specifically was deemed to be of particular importance at the time of study design, as the role of this outcome in peripheral T-cell lymphoma had not previously been assessed in a dedicated, prospective cohort of patients with peripheral T-cell lymphoma. All patients receiving at least one dose of study medication were included in the safety analysis and analysed as treated. The severity of adverse events was defined according to the National Cancer Institute Common Terminology Criteria for Adverse Events version 4.0. The worst toxicity per organ per patient was considered.

A planned subgroup analysis was done to determine the factors associated with complete response or unconfirmed complete response by treatment arm in the assessable population with the following factors: peripheral T-cell lymphoma subtype, International Prognostic Index, sex, B symptoms, age, disease stage, WHO performance status, presence of extranodal disease, and raised lactate dehydrogenase concentration.

### Statistical analysis

We calculated the patient sample size for this trial estimating that 50% of patients in the CHOP group and 70% of patients in the GEM-P group would achieve a complete response or unconfirmed complete response by CT, with an odds ratio of 2·33. We calculated that 93 patients per group were required to detect this difference with 5% significance (two-sided) and 80% power. Continuity correction was not used.

A planned formal interim analysis with 90% power to show non-inferiority for review by an independent data monitoring committee was due after the first 51 patients in each group had been assessed for end-of-treatment response; however, this milestone was not reached as the trial closed before accruing 51 patients per group. A lower 90% one-sided CI of difference in the number of patients achieving a confirmed or unconfirmed complete response between treatments was expected to be greater than 25% (ie, a non-inferiority margin of 25%).

The number of patients achieving a complete response or unconfirmed complete response at the end of study chemotherapy was reported by treatment group with 95% CI using normal approximation and compared between groups using logistic regression, with odds ratio (OR [95% CI]) for CHOP versus GEM-P. Additionally, a logistic regression model was fitted to adjust for the stratification variables, with OR (95% CI) for CHOP versus GEM-P.

In the planned subgroup analysis, we used logistic regression to assess treatment differences in responses within the predefined subgroups, with results displayed as ORs in a Forest plot. Progression events were defined as clinical or radiological documented disease progression or death from any cause. Patients recording no event were censored at the last follow-up date. Progression-free survival and overall survival were calculated from the date of randomisation until a progression or death event occurred, respectively, using Kaplan-Meier methods and compared between groups using a log-rank test. Additionally, a Cox proportional hazards model was fitted to adjust for the stratification variables and to calculate a hazard ratio (HR [95% CI]) for CHOP versus GEM-P. The ITT population was used for survival analyses.

We assessed the following factors affecting overall survival and progression-free survival using Cox regression analysis in the ITT population combining both treatment groups: age, sex, disease stage, WHO performance status, B symptoms, presence of raised lactate dehydrogenase, International Prognostic Index risk group, subtype of peripheral T-cell lymphoma, number of extranodal sites, treatment arm, local CT response after study chemotherapy, central ^18^F-FDG-PET-CT response after study chemotherapy, and autologous stem-cell transplantation following first-line treatment. For safety analyses, the proportion of patients reporting toxicities of grade 3 or worse was compared between groups using a χ^2^ test.

The data analysis was generated using Stata version 14. The trial was overseen by trial management and trial steering committees and an independent data monitoring committee. This trial is registered with ClinicalTrials.gov (NCT01719835) and the European Clinical Trials Database (EudraCT 2011-004146-18).

### Role of the funding source

The trial sponsor (The Royal Marsden Hospital, London, UK) was responsible for randomisation, data gathering, entry, and validation, reports of serious adverse events, organisation of the central histopathology review and central response assessment, statistical analysis, and production of the report. The funder of the study had no role in study design, data collection, data analysis, data interpretation, or writing of the report. The corresponding author had full access to all the data in the study and had final responsibility for the decision to submit for publication.

## Results

**Figure 1:**
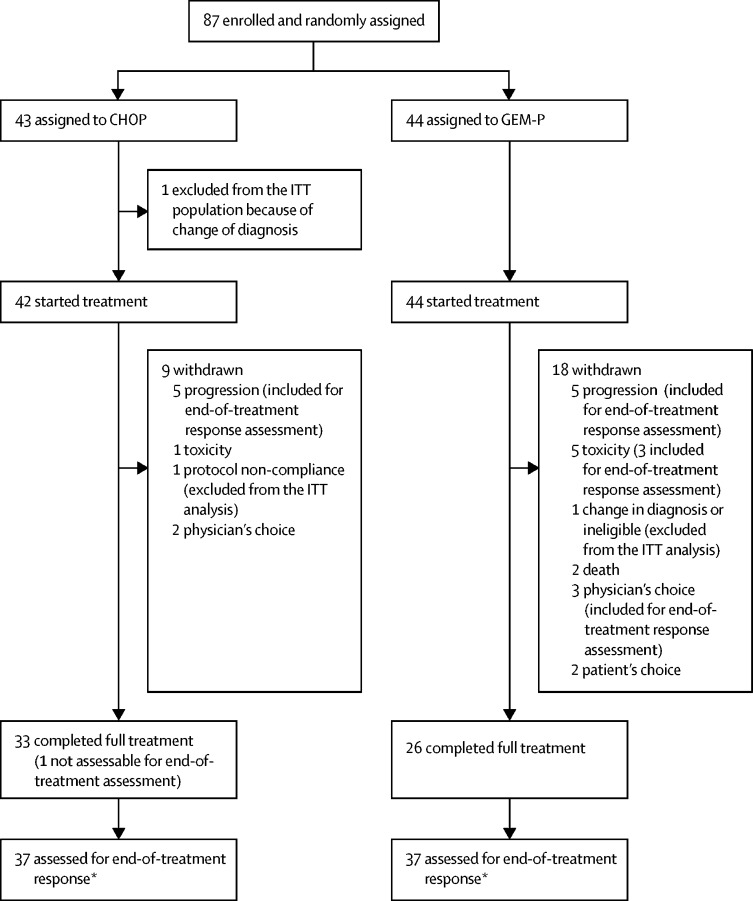
Trial profile Toxicity reasons for early study withdrawal were neutropenic sepsis (n=1) in CHOP; and tinnitus (n=2), hearing loss (n=1), peripheral neuropathy (n=1), and thrombocytopenia and anaemia (n=1) in GEM-P. Two deaths occurred in the GEM-P group, both due to lung infections. One patient chose to discontinue GEM-P and commence CHOP off-study at the time of study closure. GEM-P=gemcitabine, cisplatin, and methylprednisolone. CHOP=cyclophosphamide, doxorubicin, vincristine, and prednisolone. ITT=intention-to-treat. *Includes three patients with clinical progression.

Between June 18, 2012, and Nov 16, 2016, we randomly assigned 87 patients to study treatment, 43 to CHOP and 44 to GEM-P (figure 1). Before the formal interim analysis, interim results from a planned unmasked independent review of efficacy data by the independent data monitoring committee in November, 2016, showed that fewer patients had achieved a complete response or unconfirmed complete response with GEM-P than with CHOP. The committee concluded there was a high likelihood that GEM-P would be inferior to CHOP at the end of the trial, as indicated by the one-sided 80% CI for difference. The trial was subsequently closed to recruitment and all patients treated in the GEM-P group were offered the option to change to CHOP off-study at the time of study closure as per the recommendations of the data monitoring committee and trial management group. The last dose of study treatment on trial was administered on Feb 23, 2017.

Patient baseline and disease characteristics were well balanced between groups (table 1). All 71 patients with sufficient tissue for central histopathology review had the diagnosis of peripheral T-cell lymphoma confirmed centrally, although the subtype of peripheral T-cell lymphoma was revised for 16 patients (23%).

**Table 1:** Baseline characteristics of all randomly assigned patients

		**CHOP (six cycles; n=43)**	**GEM-P (four cycles; n=44)**
Sex			
	Men	30 (70%)	32 (73%)
	Women	13 (30%)	12 (27%)
Age (years; median IQR)		64 (54–69)	61 (50–70)
Aged >60 years		27 (63%)	24 (55%)
IPI score			
	0–1	9 (21%)	8 (18%)
	2–3	26 (60%)	25 (57%)
	4–5	8 (19%)	11 (25%)
Local histology			
	PTCL not otherwise specified	19 (44%)	18 (41%)
	ALK-negative ALCL	6 (14%)	8 (18%)
	AITL	17 (40%)	17 (39%)
	EATL	1 (2%)	1 (2%)
	Hepatosplenic γδ T-cell lymphoma	0	0
Central histology			
	PTCL not otherwise specified	11 (26%)	10 (23%)
	ALK-negative ALCL	4 (9%)	4 (9%)
	AITL	22 (51%)	17 (39%)
	EATL	1 (2%)	0
	Panniculitis-like T-cell lymphoma	0	1 (2%)
	PTCL not otherwise specified or panniculitis-like T-cell lymphoma	0	1 (2%)
	Not assessable	5	11 (25%)
Stage			
	I	1 (2%)	0
	II	8 (19%)	3 (7%)
	III	16 (37%)	16 (36%)
	IV	18 (42%)	25 (57%)
B symptoms present		26 (60%)	27 (61%)
Increased lactate dehydrogenase		27 (63%)	27 (61%)
Extranodal sites present		25 (58%)	30 (68%)
WHO performance status			
	0	17 (40%)	21 (48%)
	1	19 (44%)	18 (41%)
	2	7 (16%)	5 (11%)

Data are n (%) except where indicated otherwise. CHOP=cyclophosphamide, doxorubicin, vincristine, and prednisolone. GEM-P=gemcitabine, cisplatin, and methylprednisolone. IPI=International Prognostic Index. PTCL=peripheral T-cell lymphoma. ALK-negative ALCL=anaplastic lymphoma kinase-negative anaplastic large cell lymphoma. AITL=angioimmunoblastic T-cell lymphoma. EATL=enteropathy-associated T-cell lymphoma.

After receiving four cycles of GEM-P, one patient in the GEM-P group received an additional two cycles for persistent bone marrow infiltration before autologous stem cell transplantation. 16 patients (38%) of 42 treated with CHOP and 23 (52%) of 44 treated with GEM-P had at least one treatment delay, and 18 patients (43%) of 42 treated with CHOP and 21 (48%) of 44 treated with GEM-P had at least one dose reduction on study. Median total dose received and dose intensities for each drug by treatment group are in table 2.

**Table 2:** Total chemotherapy dose received by patients and dose intensity achieved

	**CHOP**				**GEM-P**		
	Cyclophosphamide	Doxorubicin	Vincristine	Prednisolone	Gemcitabine	Cisplatin	Methylprednisolone
Total dose received (mg)	7960 (5080–8760)	540 (342–588)	12 (7–12)	2400 (1500–3000)	18262·5 (7060–21900)	361·5 (201·5–707·5)	20 000 (7500–20 000)
Relative dose intensity	98·1 (90·5–100)	97·9 (91·9–100·0)	99·2 (85·7–100·0)	97·0 (89·7–100·0)	86·5 (69·2–95·9)	82·2 (41·6–97·4)	98·1 (83·3–100·0)

Data are median (IQR).

The proportions of CHOP and GEM-P treatments requiring G-CSF support were 135 (62%) of 219 and 70 (54%) of 130, respectively. Two patients (5%) of 43 in the CHOP group and four (9%) of 44 in the GEM-P group received prophylaxis for CNS relapse during the study: four (9%) received intrathecal methotrexate, one (2%) received intrathecal methotrexate plus methotrexate, cytarabine, and hydrocortisone, and one (2%) received unspecified therapy.

**Figure 2:**
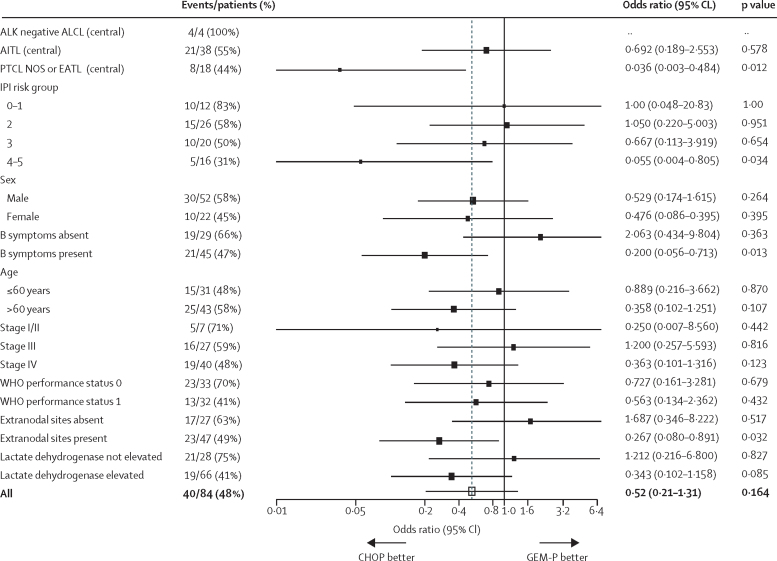
Factors potentially predictive of a complete response or unconfirmed complete response Data are from an analysis of outcomes at end of treatment. CHOP=cyclophosphamide, doxorubicin, vincristine, and prednisolone. GEM-P=gemcitabine, cisplatin, and methylprednisolone. ALK-negative ALCL= anaplastic lymphoma kinase-negative anaplastic large cell T-cell lymphoma. AITL=angioimmunoblastic T-cell lymphoma. PTCL NOS=peripheral T-cell lymphoma not otherwise specified. EATL=enteropathy-associated T-cell lymphoma. IPI=International Prognostic Index. ··=data not obtainable.

Investigator-assessed end of treatment response by contrast-enhanced CT was available for 74 patients (37 in each group; table 3). At a median follow-up of 27·4 months (IQR 16·6–38·4), 23 patients (62%) of 37 assigned to CHOP had achieved a complete response or unconfirmed complete response compared with 17 (46%) of 37 assigned to GEM-P (OR 0·52, 95% CI 0·21–1·31; p=0·164; adjusted OR for stratification factors 0·53 (0·19–1·46, p=0·22). Given the higher proportion of patients with stage IV disease in the GEM-P group versus CHOP, the logistic regression was adjusted for stage as well as for stratification variables, but this did not make any difference to the complete response outcome by randomisation result (OR 0·50, 95% CI 0·18–1·40; p=0·19). Sensitivity analyses of the primary endpoint in both the ITT and per-protocol populations showed no differences between the treatment groups (appendix p 3). The inferiority of complete response outcomes with GEM-P was consistent across subgroups (figure 2).

**Table 3:** End of treatment response by CT

	**CHOP (six cycles; n=43)**	**GEM-P (four cycles; n=44)**
Overall response	28 (75·7)	25 (67·6)
Complete response or unconfirmed complete response*	23 (62·2)	17 (45·9)
Partial response	5 (13·5)	8 (21·6)
Stable disease	2 (5·4)	3 (8·1)
Progressive disease	4 (10·8)	6 (16·2)
Progressive disease assessed clinically	3 (8·1)	3 (8·1)
Not done or not assessable	6	7

Data are n (%) of those who had an assessment. CHOP=cyclophosphamide, doxorubicin, vincristine, and prednisolone. GEM-P=gemcitabine, cisplatin, and methylprednisolone.

* p=0·164.

After chemotherapy, four (9%) of 43 patients in the CHOP group underwent radiotherapy; no patients in the GEM-P arm received radiotherapy. Stem cells were collected for 16 patients (37%) of 43 in the CHOP group and 15 (34%) of 44 in the GEM-P group, with 11 (26%) in CHOP and 13 (30%) in GEM-P proceeding to autologous stem-cell transplantation in first remission. Second-line chemotherapy was administered to 15 patients (35%) of 43 in the CHOP group and 20 (45%) of 44 in the GEM-P group.

Survival was assessed in the ITT population (84 patients [97%] of 87). Three patients (two in the CHOP group, one in the GEM-P group) were excluded from the ITT population following treatment assignment, two because of a change in diagnosis from peripheral T-cell lymphoma not otherwise specified to Hodgkin's lymphoma after randomisation and a third patient was deemed to be ineligible following treatment assignment because of cardiac impairment. 2-year progression-free survival was 38·0% (95% CI 22·9–52·9) in the GEM-P group and 36·6% (21·4–52·0) in the CHOP group (HR 1·07, 95% CI 0·61–1·85, p=0·82; appendix p 4). Two patients (2%) of 84 had CNS relapses (both isolated CNS recurrences); both patients had peripheral T-cell lymphoma not otherwise specified, were treated with CHOP, and developed progression in the CNS after cycle 1 and cycle 5, respectively. 2-year overall survival was 63·9% (95% CI 45·7–77·4) in the GEM-P group and 51·0% (32·8–67·4) in the CHOP group (HR 0·69, 95% CI 0·35–1·38, p=0·30; appendix p 5).

Significant risk factors associated with superior overall survival in univariable analysis (appendix p 6) were a complete response or unconfirmed complete response on completion of treatment determined by CT, a complete response on completion of treatment determined by ^18^F-FDG-PET-CT, and autologous stem-cell transplantation consolidation in first remission. No factor remained independently prognostic for overall survival in multivariable analysis (appendix p7). Presence of raised lactate dehydrogenase, an International Prognostic Index score of more than 1, and presence of more than one extranodal site was associated with inferior progression-free survival in univariable analysis (appendix p 8), while patients with a complete response or unconfirmed complete response to induction determined by CT or a complete response to induction determined by ^18^F-FDG-PET-CT or undergoing autologous stem-cell transplantation consolidation in first remission had superior progression-free survival. In multivariable analysis presence of raised lactate dehydrogenase concentrations at diagnosis was independently associated with inferior progression-free survival; by contrast, attainment of a complete response by ^18^F-FDG-PET-CT to study treatment or presence of a low or low-intermediate risk international prognostic index score was independently associated with superior progression-free survival (appendix p9)

Two patients (5%) died during the study, both in the GEM-P group, from lung infections. Up to Nov 7, 2017, 33 patients (39%) of 84 had died in the ITT population, 18 (44%) of 41 in the CHOP group (13 from disease progression and five from sepsis) and 15 (35%) of 43 in the GEM-P group (nine from disease progression, three from sepsis, one cardiac event, one from ischaemic colitis and disease progression, and one from metastatic cancer).

Safety was assessed for 86 patients (99%) who received a dose of study treatment. CHOP was associated with more febrile neutropenia of all grades (table 4). GEM-P was associated with more thrombocytopenia of all grades and grade 3 or worse; however, thrombocytopenia was not associated with increased bleeding. GEM-P was also associated with more tinnitus. There were 38 serious adverse events in the CHOP group and 43 in the GEM-P group.

**Table 4:** Adverse events by treatment

	**CHOP (n=42)**			**GEM-P (n=44)**		
	Grade 1–2	Grade 3	Grade 4	Grade 1–2	Grade 3	Grade 4
Acute renal toxicity	0	0	0	1 (2%)	1 (2%)	0
Alopecia*	27 (64%)	0	0	12 (27%)	0	0
Anaemia	23 (55%)	4 (10%)	0	22 (50%)	5 (11%)	1 (2%)
Constipation	18 (43%)	0	0	20 (45%)	1 (2%)	0
Cerebrovascular accident	0	0	0	0	0	1 (2%)
Diarrhoea	15 (36%)	0	0	13 (30%)	2 (5%)	0
Dyspnoea	4 (10%)	0	0	6 (14%)	0	0
Fatigue	29 (69%)	2 (5%)	0	35 (80%)	0	0
Febrile neutropenia†	0	10 (24%)	2 (5%)	1 (2%)	3 (7%)	0
Fever	11 (26%)	3 (7%)	0	11 (25%)	2 (5%)	1 (2%)
Haemorrhage	4 (10%)	0	0	7 (16%)	0	1 (2%)
Headache*	4 (10%)	0	0	13 (30%)	0	0
Hyperglycaemia	1 (2%)	1 (2%)	0	3 (7%)	1 (2%)	0
Hypertension	1 (2%)	1 (2%)	0	3 (7%)	1 (2%)	0
Hypotension	3 (7%)	1 (2%)	0	5 (11%)	1 (2%)	0
Indigestion	14 (33%)	0	0	8 (18%)	0	0
Infection	8 (19%)	7 (17%)	0	14 (32%)	4 (9%)	2 (5%)
Infusion reaction	1 (2%)	0	0	1 (2%)	1 (2%)	0
Mood disturbance	6 (14%)	0	0	7 (16%)	1 (2%)	0
Mucositis*	20 (48%)	1 (2%)	0	11 (25%)	0	0
Nausea	17 (40%)	0	0	22 (50%)	1 (2%)	0
Neuropathy	11 (26%)	1 (2%)	0	7 (16%)	0	0
Neutropenia	5 (12%)	2 (5%)	15 (36%)	7 (16%)	5 (11%)	4 (9%)
Pulmonary embolus	0	2 (5%)	1 (2%)	0	1 (2%)	0
Pruritus	7 (17%)	0	0	9 (20%)	0	0
Skin rash	7 (17%)	0	0	12 (27%)	0	0
Thrombocytopenia†	8 (19%)	1 (2%)	3 (7%)	16 (36%)	8 (18%)	5 (11%)
Tinnitus*	1 (2%)	0	0	12 (27%)	0	0
Vomiting	12 (29%)	1 (2%)	0	13 (30%)	2 (5%)	0
Weight loss	10 (24%)	1 (2%)	0	9 (20%)	1 (2%)	0
Other toxicity	23 (55%)	5 (12%)	1 (2%)	27 (61%)	9 (20%)	2 (5%)

Data are n (%) for adverse events of grade 1–2 occurring in at least 10% of patients and all grade 3–5 events. Two patients (5%) died during the study, both in the GEM-P group, from lung infections. Other adverse events with CHOP of grade 3 or worse were superior mesenteric artery thrombosis (grade 3), muscle weakness (grade 3), insomnia (grade 3), hyponatraemia (grade 4), tumour lysis (grade 3), pain left hip and legs (grade 3), swelling in left knee (grade 3), and squamous cell carcinoma cheek (grade 3). Other adverse events with GEM-P of grade 3 or worse were right flank pain (grade 3), abdominal pain (grade 3), colonic perforation (grade 4), hip pain (grade 3), chest pain (grade 3), chest/abdominal pain (grade 3), paraneoplastic occurrence (grade 4), raised alanine transaminase (grade 3), bone pain (grade 3), dehydration (grade 3), hypokalaemia (grade 3), raised alanine transaminase (grade 3), and cellulitis (grade 3). CHOP=cyclophosphamide, doxorubicin, vincristine, and prednisolone. GEM-P=gemcitabine, cisplatin, and methylprednisolone.

* p<0.05 for all grades.

† p<0.05 for all grades and grade 3 or worse.

In the ^18^F-FDG-PET-CT substudy, 79 (91%) of 87 patients assessed had FDG-avid disease at baseline on central review of imaging. End-of-treatment response by ^18^F-FDG-PET-CT or CT was assessable in 70 patients (89%) of 79 (appendix p 10); although the incidence of complete response as determined by ^18^F-FDG-PET-CT was lower in the GEM-P group than in the CHOP group, the difference between groups was less marked than between-group differences for complete response assessment by contrast-enhanced CT. The proportion of agreement between ^18^F-FDG-PET-CT and contrast-enhanced CT for determining a complete response was 77·3%. For 40 patients with a complete response by ^18^F-FDG-PET-CT on completion of study treatment, the corresponding contrast-enhanced CT data were: 24 (60%) with a complete response, seven (18%) with an unconfirmed complete response, five (13%) with a partial response, two (5%) with stable disease, and two (5%) with progressive disease.

## Discussion

The number of complete responses or unconfirmed complete responses in the GEM-P group was non-significantly inferior to the CHOP group, indicating that the primary endpoint of the trial would not be met. A planned subgroup analysis showed that the effect was consistent across subgroups.

As part of a preplanned analysis, we also assessed the proportion of agreement between contrast-enhanced CT and ^18^F-FDG-PET-CT for determining a complete response, and found it to be around 77%. This discrepancy was probably mainly due to the presence of persistent non-FDG-avid nodes discernible on contrast-enhanced CT after chemotherapy and ^18^F-FDG-PET-CT might be better able to distinguish between a residual fibrotic mass following chemotherapy versus viable tumour in peripheral T-cell lymphoma. The rate of early withdrawal before completing study treatment for reasons other than disease progression was higher in the GEM-P group with more participants withdrawing for toxicity and by choice; there were also two deaths on treatment with GEM-P, both due to lung infections. Patients treated in the CHOP group had more febrile neutropenia than those in the GEM-P group; by contrast, patients treated with GEM-P had more thrombocytopenia of all grades and grade 3 or worse than those treated with CHOP, although this did not lead to a substantial increase in bleeding events. GEM-P was also associated with a significant increased risk of grade 1-2 tinnitus, which led two patients to withdraw early from the study.

Relative dose intensity was lower in the GEM-P group than in the CHOP group, although this might mainly reflect the dose modifications that were required with the GEM-P regimen according to blood results on the day of treatment. However, there were two separate instances where a day of GEM-P treatment was incorrectly omitted rather than delayed, resulting in two protocol violations, although it seems unlikely that this modification would have significantly negatively affected the primary endpoint assessment in the GEM-P arm. At the median follow-up of 27·4 months there was no difference in either 2-year progression-free survival or overall survival between the treatment groups. In a Cox regression analysis of the ITT population, significant risk factors associated with superior overall survival included a complete response at end of treatment determined either by CT or by ^18^F-FDG-PET-CT, and autologous stem-cell transplantation consolidation in first remission in univariable analysis. However, no factors remained independently significant for overall survival in multivariable analysis. In multivariable analysis of the factors associated with progression-free survival, presence of a raised lactate dehydrogenase concentration at presentation was prognostic of inferior progression-free survival, whereas a low or low-intermediate risk international prognostic index score or a complete response to study treatment determined by ^18^F-FDG-PET-CT was independently associated with superior progression-free survival.

The number of patients who withdrew from the study for reasons other than disease progression was higher in the GEM-P group than in the CHOP group, and consequently more patients receiving GEM-P proceeded to second-line chemotherapy. This might explain the absence of a survival difference between arms at this time, as the numbers undergoing autologous stem-cell transplantation in first remission were similar between groups, although this remains to be further investigated. However, given that the inferior incidence of complete responses and unconfirmed complete responses in the GEM-P group was not associated with inferior progression-free survival or overall survival for these patients, complete response (particularly when assessed by contrast-enhanced CT) might not be an accurate surrogate endpoint in peripheral T-cell lymphoma. Future studies in this area should therefore better investigate this point, and perhaps should be designed with primary endpoints that incorporate survival. Indeed, measuring endpoints such as 24-month event-free survival in peripheral T-cell lymphoma might be an important predictor of overall survival; one study showed that patients achieving 24-month event-free survival had superior overall survival compared with those who did not reach this milestone.^24^

Although our study closed early to recruitment, this phase 2 trial is an important addition to the prospective evidence-base for this rare subtype of non-Hodgkin lymphoma and is, to our knowledge, the third randomised trial reported with treatment-naive patients with peripheral T-cell lymphoma. All participants had the diagnosis and subtype of peripheral T-cell lymphoma confirmed centrally by two expert lymphoma pathologists; for around 23% of patients with peripheral T-cell lymphoma the subtype was revised, highlighting that there are challenges to accurately diagnosing peripheral T-cell lymphoma. Additionally, although extensive future work is necessary, our study reports, to our knowledge, the first dedicated prospective trial to assess ^18^F-FDG-PET-CT responses in patients with peripheral T-cell lymphoma, all of which were done at accredited sites, underwent central quality control, and were centrally assessed by an expert ^18^F FDG PET-CT physician. Our study also adds important data from a prospective trial cohort on the incidence of CNS relapse in peripheral T-cell lymphoma, which at the time of writing has been reported in two patients with peripheral T-cell lymphoma not otherwise specified and occurred during study treatment.

However, the limitations of our study also need to be acknowledged. Firstly, and most importantly, as the trial closed early to recruitment it was not adequately powered to assess the primary study endpoint. Another potential limitation of the study is in the interpretation of dose intensity for GEM-P; this regimen was administered weekly for 3 weeks out of 4, with the dose adjusted on each day of treatment in accordance with the blood counts on the day of treatment, which is in contrast to CHOP where treatment is administered once every 3 weeks allowing more time for count recovery.

Despite the absence of international consensus on the gold-standard therapy for previously untreated patients with peripheral T-cell lymphoma, CHOP has been adopted by many countries as the reference regimen.^25^ With the exception of ALK-positive anaplastic large cell T-cell lymphoma^26^ the outcomes with CHOP or CHOP-like therapy appear to be suboptimum, with a 5-year overall survival of only 38·5%, as reported in a meta-analysis.^5^ Attempts to surpass outcomes with CHOP have proven largely unsuccessful, except a subgroup analysis by the German High-Grade NHL Study Group that showed improved event-free survival but no overall survival benefit with CHOEP for younger patients (≤60 years) with peripheral T-cell lymphoma with normal lactate dehydrogenase concentrations treated within aggressive non-Hodgkin lymphoma clinical trials.^6^ Therefore, optimising therapy for newly diagnosed patients remains an important unmet medical need.

MDR-1/Pgp is known to be overexpressed in peripheral T-cell lymphoma^7^, ^8^ in the lymphoma cells, residual lymphocytes, and endothelium,^8^ and this might account for the reported efficacy of the nucleoside analogue gemcitabine in peripheral T-cell lymphoma, which is not a substrate of the efflux pump.^27^ The activity of intravenous gemcitabine monotherapy (1200 mg/m^2^ on days 1, 8, and 15 of a 28-day cycle) was first reported in 1998 in a phase 2 study^9^ of 13 patients with relapsed or refractory peripheral T-cell lymphoma unspecified or patients with mycosis fungoides with an objective response of 69% and complete response of 8%. Similar efficacy was shown in another study^10^ (objective response 60% and complete response 20%) in a cohort of ten patients with relapsed or refractory T-cell lymphoma using the same dosing schedule. A subsequent study in 2010 confirmed this efficacy (objective response 51%, complete response 23%) in a larger number of relapsed or refractory patients (n=39) with some durable remissions.^11^

Various combinations of gemcitabine with platinum and steroid in relapsed or refractory peripheral T-cell lymphoma have also been reported in the scientific literature with encouraging results (objective response 36%–100%) and acceptable toxicity.^12^, ^13^, ^14^, ^15^, ^16^, ^17^, ^18^, ^19^, ^20^, ^28^ Specifically, the GEM-P regimen has been assessed in three retrospective studies including patients with peripheral T-cell lymphoma, predominantly with relapsed or refractory disease, with reported objective responses of 69%–100% (complete response 19%–50%) and some durable remisisons.^14^, ^15^, ^16^ Furthermore, some novel regimens incorporating gemcitabine have been assessed in treatment-naive patients in the peripheral T-cell lymphoma setting,^4^, ^8^, ^29^, ^30^ including one randomised trial,^4^ and all have shown encouraging results for gemcitabine in this indication excepting one.^8^ In one trial, patients were assigned either to CHOP (n=51) or a combination of gemcitabine 800 mg/m^2^ on days 1 and 8, cisplatin 25 mg/m^2^ intravenously on days 1–3, prednisolone 60 mg/m^2^ orally on days 1–5, and thalidomide 200 mg orally once per day continuously (n=52); this regimen was associated with a significant improvement in the proportion of patients achieving a response, 2-year progression-free survival, and 2-year overall survival.^4^

Our study confirms the poor outcomes for patients with peripheral T-cell lymphoma in the setting of a prospective randomised trial.^5^, ^25^ Recruitment to the trial was closed early as there was strong evidence that the primary endpoint—to detect superiority of GEM-P over CHOP by a comparison of the CT-based complete responses and unconfirmed complete responses at end of treatment—would not be met, although this was not reflected in inferior progression-free survival or overall survival at 2 years in the GEM-P group. More patients also withdrew from the GEM-P group than from the CHOP group for reasons other than disease progression. The dose of cisplatin 100 mg/m^2^ administered in GEM-P was associated with more grade 1–2 tinnitus (compared with CHOP), which led three patients to withdraw early before completing study treatment; this dose therefore appears to be at the upper limit of what is tolerable in terms of ototoxicity, but further assessment of the dosing limit would be necessary to establish tolerability. Nevertheless, we have revised the dose of cisplatin administered with GEM-P at The Royal Marsden Hospital (London, UK) to a total dose of 75 mg/m^2^, in line with the dose used in other regimens containing gemcitabine and platinum,^4^, ^31^ with the expectation that this might reduce the incidence of ototoxicity.

Although our data show that GEM-P has efficacy in terms of response and survival in peripheral T-cell lymphoma, in our randomised study it was inferior to CHOP for treatment-naive patients with this disease. One possible exception for the use of this type of regimen upfront might be when front-line anthracycline-based chemotherapy is contraindicated to avoid cardiotoxicity. In conclusion, although further studies are warranted, our phase 2 randomised trial suggests that CHOP should, for the time being, remain the reference regimen for previously untreated patients with peripheral T-cell lymphoma and that GEM-P is best reserved for the relapsed and refractory setting at present.

## References

[1] Vose JM, Armitage J, Weisenburger D (2008). International peripheral T-cell and natural killer/T-cell lymphoma study: pathology findings and clinical outcomes international T-cell lymphoma project. J Clin Oncol.

[2] Pautier P, Devidas A, Delmer A (1999). Angioimmunoblastic-like T-cell non Hodgkin's lymphoma: outcome after chemotherapy in 33 patients and review of the literature. Leuk Lymphoma.

[3] Simon A, Peoch M, Casassus P (2010). Upfront VIP-reinforced-ABVD (VIP-rABVD) is not superior to CHOP/21 in newly diagnosed peripheral T cell lymphoma. Results of the randomized phase III trial GOELAMS-LTP95. Br J Haematol.

[4] Li L, Duan W, Zhang L (2017). The efficacy and safety of gemcitabine, cisplatin, prednisone, thalidomide versus CHOP in patients with newly diagnosed peripheral T-cell lymphoma with analysis of biomarkers. Br J Haematol.

[5] Abouyabis AN, Shenoy PJ, Sinha R, Flowers CR, Lechowicz MJ (2011). A systematic review and meta-analysis of regimens for peripheral T-cell lymphoma. ISRN Hematol.

[6] Schmitz N, Trümper L, Ziepert M (2010). Treatment and prognosis of mature T-cell and NK-cell lymphoma: an analysis of patients with T-cell lymphoma treated in studies of the German High-Grade Non-Hodgkin Lymphoma Study Group. Blood.

[7] Pescarmona E, Pignoloni P, Puopolo M (2001). p53 over-expression identifies a subset of nodal peripheral T-cell lymphomas with a distinctive biological profile and poor clinical outcome. J Pathol.

[8] Mahadevan D, Unger JM, Spier CM (2013). Phase 2 trial of combined cisplatin, etoposide, gemcitabine, and methylprednisolone (PEGS) in peripheral T-cell non-Hodgkin lymphoma: Southwest Oncology Group Study S0350. Cancer.

[9] Zinzani PL, Magagnoli M, Bendandi M (1998). Therapy with gemcitabine in pretreated peripheral T-cell lymphoma patients. Ann Oncol.

[10] Sallah S, Wan JY, Nguyen NP (2001). Treatment of refractory T-cell malignancies using gemcitabine. Br J Haematol.

[11] Zinzani PL, Venturini F, Stefoni V (2010). Gemcitabine as single agent in pretreated T-cell lymphoma patients: evaluation of the long-term outcome. Ann Oncol.

[12] Chau I, Harries M, Cunningham D (2003). Gemcitabine, cisplatin and methylprednisolone chemotherapy (GEM-P) is an effective regimen in patients with poor prognostic primary progressive or multiply relapsed Hodgkin's and non-Hodgkin's lymphoma. Br J Haematol.

[13] Emmanouilides C, Colovos C, Pinter-Brown L (2004). Pilot study of fixed-infusion rate gemcitabine with cisplatin and dexamethasone in patients with relapsed or refractory lymphoma. Clin Lymphoma.

[14] Ng M, Waters J, Cunningham D (2005). Gemcitabine, cisplatin and methylprednisolone (GEM-P) is an effective salvage regimen in patients with relapsed and refractory lymphoma. Br J Cancer.

[15] Arkenau H-T, Chong G, Cunningham D (2007). Gemcitabine, cisplatin and methylprednisolone for the treatment of patients with peripheral T-cell lymphoma: the Royal Marsden Hospital experience. Haematologica.

[16] Yim KL, Ashley S (2012). Assessment of gemcitabine, cisplatin and methylprednisolone (GEM-P) combination treatment for non-Hodgkin T cell lymphoma. Med Oncol.

[17] Dong M, He X, Liu P (2013). Gemcitabine-based combination regimen in patients with peripheral T-cell lymphoma. Med Oncol.

[18] Park BB, Kim WS, Suh C (2015). Salvage chemotherapy of gemcitabine, dexamethasone, and cisplatin (GDP) for patients with relapsed or refractory peripheral T-cell lymphomas: a consortium for improving survival of lymphoma (CISL) trial. Ann Hematol.

[19] Qi F, Dong M, He X (2017). Gemcitabine, dexamethasone, and cisplatin (GDP) as salvage chemotherapy for patients with relapsed or refractory peripheral T cell lymphoma-not otherwise specified. Ann Hematol.

[20] Skamene T, Crump M, Savage KJ (2017). Salvage chemotherapy and autologous stem cell transplantation for peripheral T-cell lymphoma: a subset analysis of the Canadian Cancer Trials Group LY.12 randomized phase 3 study. Leuk Lymphoma.

[21] Swerdlow SH, Campo E, Harris NL, Jaffe ES, Pileri SA, Stein H (2008). WHO classification of tumours of haematopoistic and lymhphoid tissues.

[22] Cheson BD, Horning SJ, Coiffier B (1999). Report of an international workshop to standardize response criteria for non-Hodgkin's lymphomas. J Clin Oncol.

[23] Cheson BD, Pfistner B, Juweid ME (2007). Revised response criteria for malignant lymphoma. J Clin Oncol.

[24] Maurer MJ, Ellin F, Srour L (2017). International assessment of event-free survival at 24 months and subsequent survival in peripheral T-cell lymphoma. J Clin Oncol.

[25] Ellin F, Landstr J, Jerkeman M, Relander T (2015). Real-world data on prognostic factors and treatment in peripheral T-cell lymphomas: a study from the Swedish Lymphoma Registry. Blood.

[26] Savage KJ, Harris NL, Vose JM (2008). ALK- anaplastic large-cell lymphoma is clinically and immunophenotypically different from both ALK + ALCL and peripheral T-cell lymphoma, not otherwise specified: report from the international peripheral T-cell lymphoma project. Blood J.

[27] Bergman AM, Pinedo HM, Talianidis I (2003). Increased sensitivity to gemcitabine of P-glycoprotein and multidrug resistance-associated protein-overexpressing human cancer cell lines. Br J Cancer.

[28] Yao Y, Tang Y, Zhu Q (2013). Gemcitabine, oxaliplatin and dexamethasone as salvage treatment for elderly patients with refractory and relapsed peripheral T-cell lymphoma. Leuk Lymphoma.

[29] Kim JG, Sohn SK, Chae YS (2006). CHOP plus etoposide and gemcitabine (CHOP-EG) as front-line chemotherapy for patients with peripheral T cell lymphomas. Cancer Chemother Pharmacol.

[30] Jia B, Hu S, Yang J (2016). Comparison of gemcitabin, cisplatin, and dexamethasone (GDP), CHOP, and CHOPE in the first-line treatment of peripheral T-cell lymphomas. Hematology.

[31] Crump M, Kuruvilla J, Couban S (2014). Randomized comparison of gemcitabine, dexamethasone, and cisplatin versus dexamethasone, cytarabine, and cisplatin chemotherapy before autologous stem-cell transplantation for relapsed and refractory aggressive lymphomas: NCIC-CTG LY.12. J Clin Oncol.

