# Supplementary material for: CHOP versus GEM-P in previously untreated patients with peripheral T-cell lymphoma (CHEMO-T): a phase 2, multicentre, randomised, open-label trial
Source: Lancet Haematol. 2018 Apr 24;5(5):e190–200. doi: 10.1016/S2352-3026(18)30039-5 (PMC5946805; doi:10.1016/S2352-3026(18)30039-5)
Supplement: Supplementary appendix [file mmc1.pdf]

THE LANCET
Haematology

Supplementary appendix

This appendix formed part of the original submission and has been peer reviewed. We post it as supplied by the authors.

Supplement to: Gleeson M, Peckitt C, Mong To Y, et al. CHOP versus GEM-P in previously untreated patients with peripheral T-cell lymphoma (CHEMO-T): a phase 2, multicentre, randomised, open-label trial. *Lancet Haematol* 2018; **5:** e190–200.

Supplementary Appendix

Supplementary Table 1: CHEMO-T recruiting centres

Site No.	Site	P.I.	Patients recruited (N)
13	Blackpool Victoria, Blackpool Teaching Hospitals NHS Foundation Trust	Marian Macheta	9
1	The Royal Marsden NHS Foundation Trust	David Cunningham	8
5	Churchill Hospital, Oxford University Hospitals NHS Foundation Trust	Graham Collins	6
3	The Christie NHS Foundation Trust	John Radford	5
18	New Victoria Glasgow, NHS Greater Glasgow and Clyde	Alistair Hart	4
43	Royal Cornwall Hospitals NHS Trust	Adam Forbes	4
20	The Royal Liverpool and Broadgreen University Hospitals NHS Trust	Nagesh Kalakonda	4
42	Barts Health NHS Trust	Silvia Montoto	3
32	Gloucestershire Hospitals NHS Foundation Trust	Kim Benstead	3
19	Royal Hallamshire Hospital, Sheffield Teaching Hospitals NHS Foundation Trust	Nick Morley	3
24	Sandwell and West Birmingham Hospitals NHS Trust	Yasmin Hasan	3
15	The Beatson West of Scotland Cancer Centre	Pam McKay	3
22	University College London Hospitals NHS Foundation Trust	Kirit Ardeshna	3
9	Guy's and St Thomas' NHS Foundation Trust	Paul Fields	2
36	Lewisham Hospital, Lewisham and Greenwich NHS Trust	Naheed Mir	2
31	Nottingham University Hospitals NHS Trust	Christopher Fox	2
23	The Ipswich Hospital NHS Trust	Andrew Hodson	2
11	The Royal Devon and Exeter NHS Foundation Trust	Claudius Rudin	2
12	Torbay and South Devon NHS Foundation Trust	Deborah Turner	2
29	United Lincolnshire Hospitals NHS Trust	Gamal Sidra	2
14	Yeovil District Hospital NHS Foundation Trust	Belinda Austen	2
47	Eastern Health Box Hill Australia	Eliza Hawkes	1

Supplementary Table 1: CHEMO-T recruiting centres (continued)

Site No.	Site	P.I.	Patients recruited (N)
2	Mount Vernon Cancer Centre, East and North Hertfordshire NHS Trust	Peter Hoskin	1
44	Musgrove Park Hospital, Taunton and Somerset NHS Foundation Trust	Belinda Austen	1
25	New Cross Hospital, The Royal Wolverhampton NHS Trust	Supratik Basu	1
28	Norfolk and Norwich University Hospitals NHS Foundation Trust	Nimish Shah	1
35	Portsmouth Hospitals NHS Trust	Ann O'Callaghan	1
30	Royal Surrey County Hospital NHS Foundation Trust	Johannes de Vos	1
21	Royal Sussex County Hospital, Brighton and Sussex University Hospitals NHS Trust	Andrew Webb	1
8	Southampton General Hospital, University Hospital Southampton NHS Foundation Trust	Peter Johnson	1
6	St James', The Leeds Teaching Hospitals NHS Trust	Rod Johnson	1
4	The Royal Free NHS Foundation Trust	Kate Cywnarski	1
10	The Royal Oldham Hospital, The Pennine Acute Hospitals NHS Trust	Allameddine Allameddine	1
40	University Hospital of Wales, Cardiff and Vale University Health Board	Clare Rowntree	1

P.I: Principle Investigator

Supplementary Table 2: CT end of treatment response (intention to treat analysis)

	CHOP (6 cycles) N=41		GEM-P (4 cycles) N=43	
	N	(%)	N	(%)
PR / SD / PD	18	43.9	26	60.5
CR / CRu	23	56.1<br>95% CI: 40.9 - 71.3	17	39.5<br>95% CI: 24.9 - 54.1

Odds Ratio: 0.51 (0.21 – 1.22), p=0.13

Adjusted Odds Ratio (for stratification factors (IPI and Histology)): 0.50 (0.20 – 1.28), p=0.15

Supplementary Table 3: CT end of treatment response (per protocol analysis)

	CHOP (6 cycles) N=33		GEM-P (4 cycles) N=26	
	N	(%)	N	(%)
PR / SD / PD	10	30.3	11	42.3
CR / CRu	23	69.7<br>95% CI: 54.0 – 85.4	15	57.7<br>95% CI: 38.7 – 76.7

Odds Ratio: 0.59 (0.20 – 1.74), p=0.34

Adjusted Odds Ratio (for stratification factors (IPI and Histology)): 0.59 (0.18 – 1.94), p=0.38

PR: partial response; SD: stable disease; PD: progressive disease; CR: complete response; CRu: unconfirmed complete response; CI: confidence interval; IPI: International Prognostic Index

Supplementary Figure 1: Progression-free survival (A) and overall survival (B) according to treatment arm

(A)

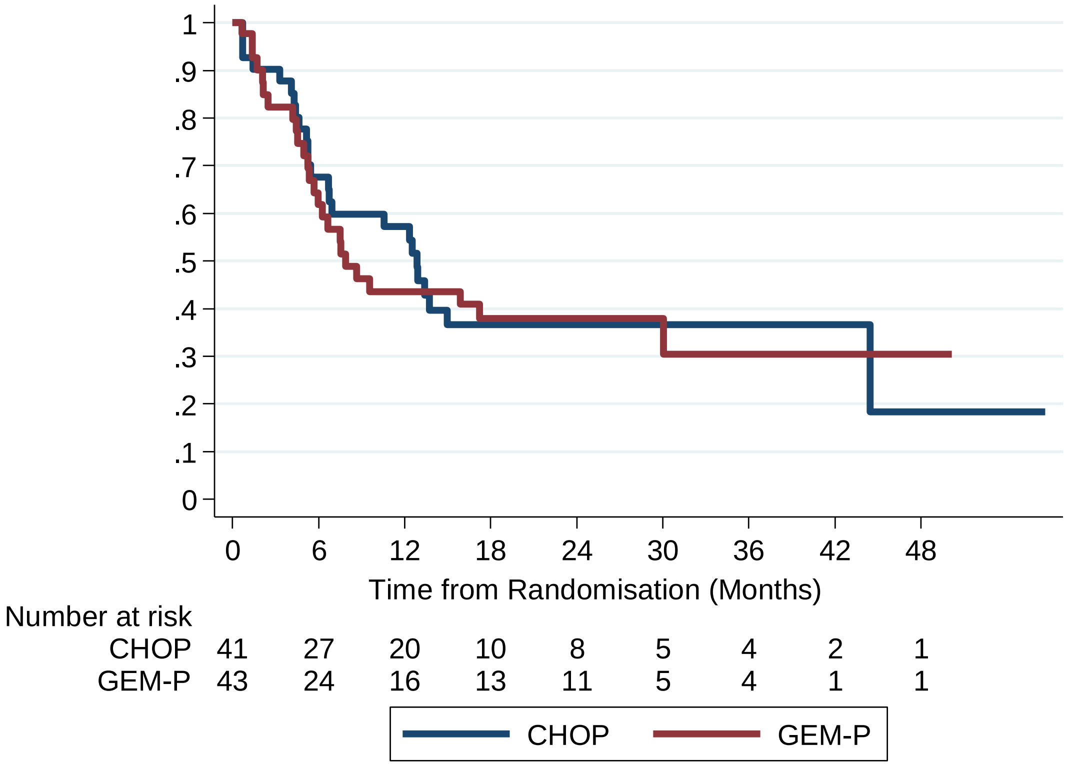

(B)

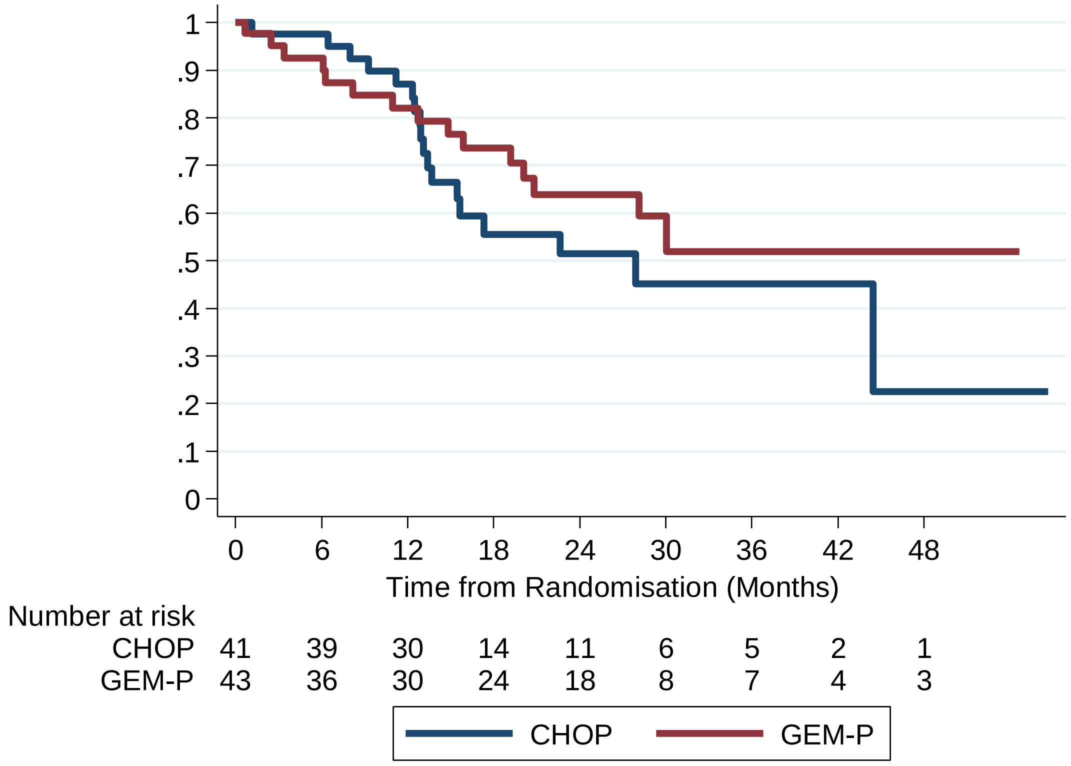
1
.9
.8
.7
.6
.5
.4
.3
.2
.1
0
0 6 12 18 24 30 36 42 48
Time from Randomisation (Months)
Number at risk
CHOP 41 39 30 14 11 6 5 2 1
GEM-P 43 36 30 24 18 8 7 4 3
CHOP
GEM-P

Supplementary Table 4: Univariable analysis of factors associated with overall survival

	N	2-yr OS	2-yr OS 95% CI	HR	HR 95% CI	p-value
**Age**						
≤ 60 years	34	66.6	45.0 - 81.3			
> 60 years	50	53.1	36.7 - 67.0	1.95	0.91 – 4.21	0.087
**Gender**						
Male	61	59.3	44.4 – 71.5			
Female	23	56.4	30.7 - 75.7	0.97	0.45 – 2.09	0.936
**Stage**						
I / II	11	90.0	47.3-98.5			
III / IV	73	54.3	40.8-66.1	2.31	0.55 - 9.64	0.252
**PS**						
0 / 1	73	59.2	45.1 – 70.8			
≥ 2	11	51.1	19.2 - 76.2	1.42	0.58 – 3.47	0.439
**B symptoms**						
Absent	33	66.4	44.8-81.1			
Present	51	53.2	36.9-67.1	1.32	0.64 – 2.73	0.453
**LDH**						
Normal	34	64.5	43.7 – 79.2			
Elevated	50	53.6	36.8 – 67.8	1.19	0.58 – 2.43	0.633
**IPI**						
Low	16	93.3	61.3 – 99.0	1.0		(0.139)
Low-intermediate	29	53.5	30.2 - 70.7	3.95	0.88 – 17.7	0.073
High-intermediate	21	42.4	17.0 – 66.0	6.12	1.3 – 28.2	**0.020**
High	18	50.0	23.9 – 71.5	4.23	0.91 – 19.7	0.066
**Subtype (central)**						
ALK negative ALCL	7	50.0	11.1 - 80.4	1.0		
AITL	39	64.0	44.9 – 78.0	0.83	0.24 - 2.86	0.764
PTCL NOS/Other	23	55.5	29.5 – 75.3	0.99	0.27 – 3.68	0.988
**Extranodal sites**						
0 – 1	58	64.4	48.9 - 76.3			
> 1	26	41.9	18.7 – 62.4	1.55	0.76 – 3.17	0.227
**Chemotherapy**						
CHOP	41	51.5	32.8 – 67.4			
GEM-P	43	63.9	45.7 – 77.4	0.69	0.35 – 1.38	0.299
**CT response (local)**						
No CR	44	44.1	26.5 – 60.4			
CR	40	71.7	53.5 – 83.8	0.44	0.21 – 0.89	**0.022**
**PET response (central)**						
No CR	28	45.7	23.1 – 65.8			
CR	42	70.7	53.3 – 82.6	0.42	0.20 – 0.90	**0.027**
**ASCT in first-line**						
No	60	50.8	35.0 – 64.6			
Yes	24	73.2	50.0 – 87.0	0.33	0.14 - 0.81	**0.015**

OS: overall survival; CI: confidence interval; HR: hazard ratio; PS: performance status; LDH: lactate dehydrogenase; IPI: International Prognostic Index; ALK negative ALCL: Anaplastic lymphoma kinase negative anaplastic large cell lymphoma; AITL: angioimmunoblastic T-cell lymphoma; PTCL NOS: peripheral T-cell lymphoma not otherwise specified; CHOP: cyclophosphamide, doxorubicin, vincristine, prednisolone; GEM-P: gemcitabine, cisplatin, methylprednisolone; CT: computed tomography; PET: positron emission tomography; ASCT: autologous stem cell transplant.

Supplementary Table 5: Multivariable analysis of factors associated with overall survival

	HR	HR 95% CI	p-value
**IPI**			
Low (0-1)	1.0		(0.175)
Low - intermediate (2)	4.86	0.56 – 42.2	0.152
High - intermediate (3)	8.25	0.93 – 73.2	0.058
High (4-5)	3,01	0.31 – 29.2	0.341
**Subtype (central)**			
ALK negative ALCL	1.0		(0.866)
AITL	0.69	0.17 – 2.79	0.602
PTCL NOS /Other	0.68	0.14 – 3.34	0.634
**CT response (local)**			
No CR	1.0		
CR	1.42	0.45 – 4.52	0.551
**PET response (central)**			
No CR	1.0		
CR	0.50	0.16 – 1.53	0.224
**ASCT in first-line**			
No	1.0		
Yes	0.61	0.20 – 1.89	0.391

HR: hazard ratio; CI: confidence interval; IPI: International Prognostic Index; ALK negative ALCL: Anaplastic lymphoma kinase negative anaplastic large cell lymphoma; AITL: angioimmunoblastic T-cell lymphoma; PTCL NOS: peripheral T-cell lymphoma not otherwise specified; CT: computed tomography; PET: positron emission tomography; ASCT: autologous stem cell transplant.

Supplementary Table 6: Univariable analysis of factors associated with progression-free survival

	N	2-yr PFS	2-yr PFS 95% CI	HR	HR 95% CI	p-value
**Age**						
≤ 60 years	34	45.4	27.3 – 61.9			
> 60 years	50	32.7	19.7 – 46.4	1.28	0.71 – 2.30	0.412
**Gender**						
Male	61	40.4	27.2 – 53.1			
Female	23	30.4	12.8 – 50.1	1.17	0.64 – 2.14	0.616
**Stage**						
I / II	11	64.0	22.6 – 87.5			
III / IV	73	33.7	22.6 – 45.0	2.72	0.84 – 8.75	0.093
**PS**						
0 / 1	73	38.1	26.3 – 49.7			
≥ 2	11	36.4	11.2 – 62.7	0.89	0.41 – 1.93	0.772
**B symptoms**						
Absent	33	40.6	23.1 – 57.4			
Present	51	35.2	21.6 – 49.1	1.15	0.64 – 2.04	0.642
**LDH**						
Normal	34	59.4	39.6 – 74.6			
Elevated	50	23.2	12.2 – 36.3	3.14	1.65 – 5.96	**<0.001**
**IPI**						
Low	16	84.9	51.2 – 96.0	1.0		**(0.002)**
Low – intermediate	29	38.1	19.7 – 56.3	7.13	1.64 – 30.9	**0.009**
Intermediate - high	21	10.6	1.8 – 28.6	15.2	3.54 – 68.0	**<0.001**
High	18	27.8	10.1 – 48.9	8.31	1.88 – 36.7	**0.005**
**Subtype (central)**						
ALK negative ALCL	7	42.9	9.8 – 73.4	1.0		
AITL	39	39.5	24.2 – 54.4	1.04	0.36 – 2.99	0.947
PTCL NOS/Other	23	30.0	11.7 – 50.8	1.24	0.40 – 3.78	0.709
**Extranodal sites**						
0 – 1	58	45.9	31.8 – 58.9			
> 1	26	19.3	6.6 – 36.9	1.99	1.13 – 3.52	**0.017**
**Chemotherapy**						
CHOP	41	36.7	21.4 – 52.0			
GEM-P	43	38.0	22.29 – 52.9	1.07	0.61 – 1.86	0.815
**CT response (local)**						
No CR	44	21.6	10.1 – 35.9			
CR	40	55.0	37.9 – 69.2	0.28	0.16 – 0.51	**<0.001**
**PET response (central)**						
No CR	28	15.0	4.7 – 30.8			
CR	42	56.3	39.3 – 70.2	0.20	0.10 – 0.38	**<0.001**
**ASCT in first-line**						
No	60	26.7	15.3 – 39.4			
Yes	24	61.9	39.4 – 78.1	0.27	0.13 – 0.57	**0.001**

PFS: progression-free survival; CI: confidence interval; HR: hazard ratio; PS: performance status; LDH: lactate dehydrogenase; IPI: International Prognostic Index; ALK negative ALCL: Anaplastic lymphoma kinase negative anaplastic large cell lymphoma; AITL: angioimmunoblastic T-cell lymphoma; PTCL NOS: peripheral T-cell lymphoma not otherwise specified; CHOP: cyclophosphamide, doxorubicin, vincristine, prednisolone; GEM-P: gemcitabine, cisplatin, methylprednisolone; CT: computed tomography; PET: positron emission tomography; ASCT: autologous stem cell transplant.

Supplementary Table 7: Multivariable analysis of factors associated with progression-free survival

	HR	HR 95% CI	p-value
**LDH**			
Normal	1.0		
Elevated	3.11	1.06 – 9.13	**0.038**
**IPI**			
Low (0-1)	1.0		**(0.006)**
Low - intermediate (2)	9.34	1.16 – 75.5	**0.036**
High - intermediate (3)	8.68	0.98 – 77.3	0.053
High (4-5)	1.86	0.18 – 19.3	0.602
**Subtype**			
ALK negative ALCL	1.0		(0.523)
AITL	0.47	0.12 – 1.80	0.269
PTCL NOS /Other	0.56	0.12 – 2.51	0.446
**Extranodal sites**			
0 – 1	1.0		
> 1	2.51	0.94 – 6.70	0.066
**CT Response (local)**			
No CR	1.0		
CR	0.77	0.31 – 1.94	0.578
**PET response (central)**			
No CR	1.0		
CR	0.16	0.06 – 0.41	**<0.001**
**ASCT in first-line**			
No	1.0		
Yes	0.52	0.20 – 1.31	0.165

HR: hazard ratio; CI: confidence interval; LDH: lactate dehydrogenase; IPI: International Prognostic Index; ALK negative ALCL: Anaplastic lymphoma kinase negative anaplastic large cell lymphoma; AITL: angioimmunoblastic T-cell lymphoma; PTCL NOS: peripheral T-cell lymphoma not otherwise specified; CT: computed tomography; PET: positron emission tomography; ASCT: autologous stem cell transplant.

Supplementary Table 8: End of treatment response by ^{18}F FDG PET-CT (intention to treat analysis)

	CHOP (6 cycles) N=41		GEM-P (4 cycles) N=43	
	**N**	**(%)***	**N**	**(%)***
**CR**	**23**	**64**	**19**	**56**
**PR**	**6**	**17**	**9**	**26**
**SD**	**0**	**0**	**1**	**3**
**PD**	**3**	**8**	**1**	**3**
**PD (assessed clinically)**	**4**	**11**	**4**	**12**
**Not done**	**5**		**9**	

***** Percentage out of those who had an assessment

ITT: Intention to treat; CR: complete response; PR: partial response; SD: stable disease; PD: progressive disease
